# Associations between superoxide dismutase, malondialdehyde and all-cause mortality in older adults: a community-based cohort study

**DOI:** 10.1186/s12877-019-1109-z

**Published:** 2019-04-15

**Authors:** Chen Mao, Jin-Qiu Yuan, Yue-Bin Lv, Xiang Gao, Zhao-Xue Yin, Virginia Byers Kraus, Jie-Si Luo, Choy-Lye Chei, David Bruce Matchar, Yi Zeng, Xiao-Ming Shi

**Affiliations:** 1 Department of Epidemiology, School of Public Health, Southern Medical University, Guangzhou, Guangdong China; 2 Scientific Research & International Exchange Department, The Seventh Affiliated Hospital, Sun Yat-sen University, Shenzhen, Guangdong China; 3 School of Public Health and Primary Care, Faculty of Medicine, The Chinese University of Hong Kong, Hong Kong, China; 4 National Institute of Environmental Health, Chinese Center for Disease Control and Prevention, #7 Panjiayuan Nanli, Chaoyang, Beijing, 100021 China; 5 Nutritional Epidemiology Lab, Pennsylvania State University, Philadelphia, PA USA; 6 Division of Non-Communicable Disease Control and Community Health, Chinese Center for Disease Control and Prevention, Beijing, China; 7 Duke Molecular Physiology Institute and Division of Rheumatology, Department of Medicine, Duke University School of Medicine, Durham, North Carolina USA; 8 Program in Health Services and Systems Research, Duke-NUS Graduate Medical School, Singapore, Singapore; 9 Center for Study of Healthy Aging and Development Studies, Peking University, Beijing, China; 10 Center for the study of Aging and Human Development and the Geriatric Division of School of Medicine, Duke University, Durham, North Carolina USA; 11 Center for Study of Healthy Aging and Development Studies, Peking University, Beijing, China

**Keywords:** Superoxide dismutase, Malondialdehyde, Mortality, Aging, Cohort

## Abstract

**Background:**

Oxidative stress is an important theory of aging but population-based evidence has been lacking. This study aimed to evaluate the associations between biomarkers of oxidative stress, including plasma superoxide dismutase (SOD) activity and malondialdehyde (MDA), with all-cause mortality in older adults.

**Methods:**

This is a community-based cohort study of 2224 participants (women:1227, median age: 86 years). We included individuals aged 65 or above and with plasma SOD activity and/or MDA tests at baseline. We evaluated the hazard ratios (HRs) and 95% confidence intervals (CIs) by multivariable Cox models.

**Results:**

We documented 858 deaths during six years of follow-up. There was a significant interaction effect of sex with the association between SOD activity and mortality (*P* < 0.001). Compared with the lowest quintile, the risk of all-cause mortality was inversely associated with increasing quintiles of plasma SOD activity in women(*P*-trend< 0.001), with adjusted HRs for the second through fifth quintiles of 0.73 (95% CI 0.53–1.02), 0.52(95% CI 0.38–0.72), 0.53(95% CI 0.39–0.73), and 0.48(95% CI 0.35–0.66). There were no significant associations between SOD activity and mortality in men (*P*-trend = 0.64), and between MDA and mortality in all participants (*P*-trend = 0.79).

**Conclusions:**

Increased activity of SOD was independently associated with lower all-cause mortality in older women but not in men. This epidemiological study lent support for the free radical/oxidative stress theory of aging.

**Electronic supplementary material:**

The online version of this article (10.1186/s12877-019-1109-z) contains supplementary material, which is available to authorized users.

## Background

Aging is the accumulation process of diverse deleterious changes in the cells and tissues with advancing age, leading to increased risk of disease and death [1]. The free radical theory proposes that aging is the cumulative result of oxidative damage to the cells and tissues of the body that arises primarily as a result of aerobic metabolism [2]. Substantial correlative evidence suggests a link of oxidative stress, an imbalance between the production and degradation of reactive oxygen species (ROS), to aging [3]. Severe oxidative stress produces excessive ROS, such as superoxide, hydroxyl, and hydrogen peroxide, which may result in damage to DNA, proteins and other cell injuries [4, 5]. The body encloses a complex antioxidant defense grid that collectively acts against free radicals. The first line defense antioxidants included superoxide dismutase (SOD), catalase and glutathione peroxidase, which play an indispensable in the entire defense strategy of antioxidants [6]. SOD is the first detoxification enzyme and most powerful antioxidant in the cell [6]. In mammals, there are three forms of SOD: SOD1 or the Cu/Zn-SOD, SOD2 or the Mu-SOD, and SOD3 or the extracellular SOD [7]. In *Saccharomyces cerevisiae* [8, 9] and Drosophila models [9, 10], SOD may protect against oxidative damage and extend life span. In addition to ROS, oxidative stress may also induce uncontrolled lipid peroxidation, which in turn, can result in cell injuries via DNA damage and directly inhibit proteins [11, 12]. Malondialdehyde (MDA) is a stable end product of lipid peroxidation and therefore can be used as an indirect measure of the cumulative lipid peroxidation. Both SOD and MDA have been considered as biological markers of oxidative stress [13].

Although in vitro data and studies in animal models have suggested that SOD and MDA may be associated with aging, epidemiologic evidence regarding their associations with mortality remains sparse. A cohort of 507 healthy residents indicated that high serum SOD activity was associated with protective effects against mortality from cancer [14]. This finding was consistent with a nested case-control study of 3653 participants [15]. In addition, blood MDA level has been shown to be associated with mortality in patients with HIV [16], chronic heart failure [17], and breast cancer [18]. However, epidemiologic studies investigating the associations of blood SOD activity, or MDA with all-cause mortality in community-dwelling older adults are still lacking. In this study, we prospectively evaluated these associations using the datasets from the Chinese Longitudinal Healthy Longevity Survey (CLHLS) in longevity areas.

## Methods

### Design, study setting, and participants

**Fig. 1:**
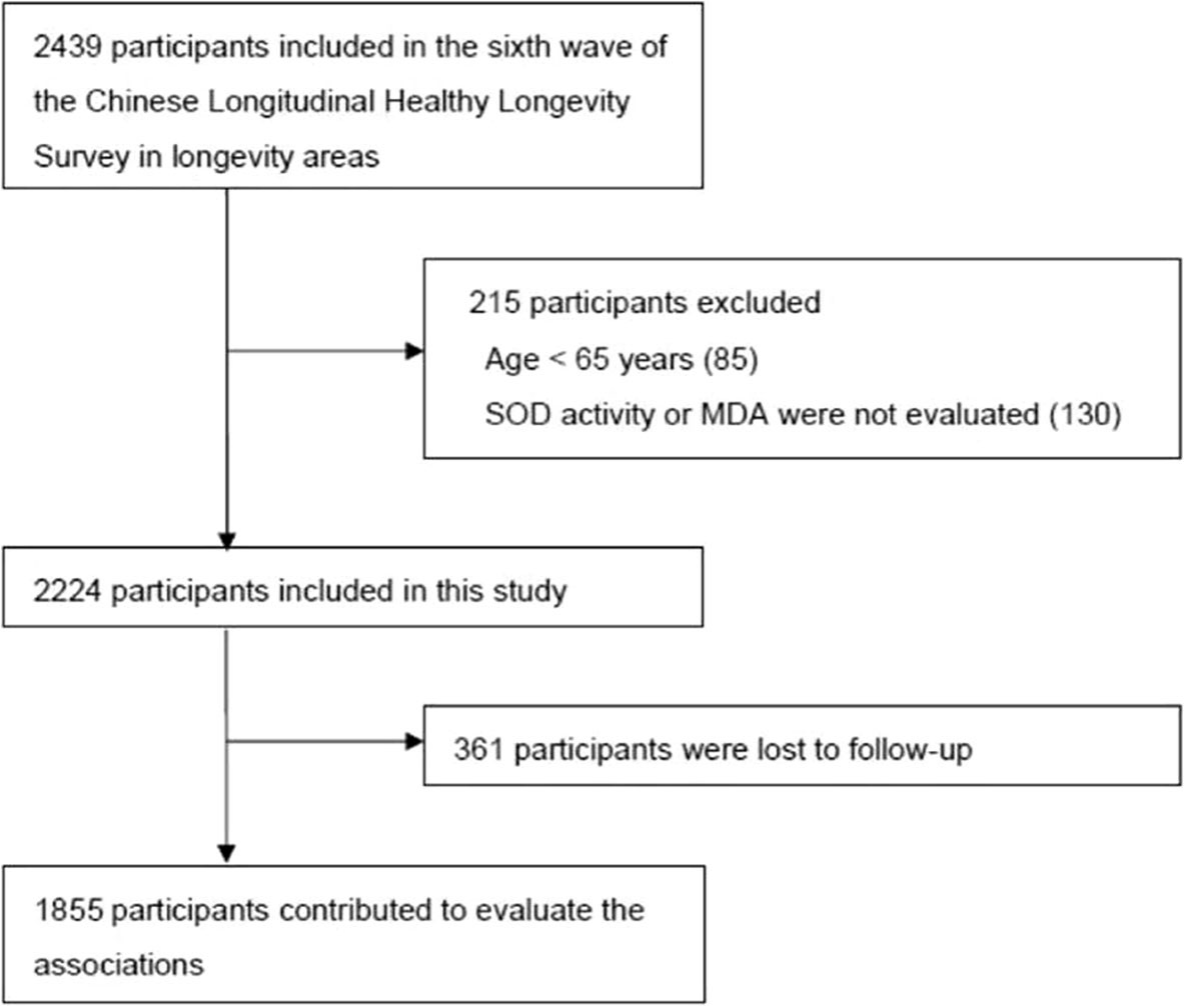
Flowchart of participant enrolment SOD: Superoxide dismutase, MDA: Malondialdehyde.

This is a prospective, community-based cohort study with a 6-year follow-up (2011–2017). Participants were recruited from the sixth wave (2011) of CLHLS in eight longevity areas, including Chen Mai county (in Hainan province), Yong Fu county (in Guangxi province), Ma Yang county (in Hunan province), Zhong Xiang city (in Hubei province), Xia Yi county (in He Nan province), San Shui city (in Guangdong province), and Lai Zhou city (in Shandong province), and Ru Dong county (in Jiangsu province). These areas represented 3/4 of the longevity areas selected by the Chinese Society of Gerontology in 2014. We included all older adults aged 65 or above and with plasma SOD activity and/or MDA tests at baseline. Overall, 2224 older adults were included, of which 361 were lost to follow-up (see the flowchart of participant enrolment in Fig. 1). More details of the CLHLS study have been described elsewhere [19]. The study was approved by the Ethics Committee of Peking University and Duke University. Informed consent was obtained from all individual participants included in the study.

### Laboratory methods

Medical professionals collected about 5 ml of venous blood samples from the older adults using blood collection tubes and heparin anticoagulant. Samples were shipped to Beijing at − 80 degrees Celsius and analyzed centrally. We tested the SOD activity with the T-SOD assay kit (Nanjing Jiangcheng Bioengineering Institute, Jiangsu, China) based on the hydroxylamine method [20]. MDA was tested with the MDA assay kit (Nanjing Jiangcheng Bioengineering Institute, Jiangsu, China) based on thiobarbituric acid method [21]. Blood biochemistry tests, including lipid profile, fasting blood glucose, high sensitivity c-reactive protein, and uric acid were analyzed by clinical chemistry analyzer (Hitachi 7180, Japan).

### Assessment of covariates

Based on a review of the literature, we selected covariates that may confound the relationship between blood SOD activity, MDA and mortality. We collected covariate information from the structured questionnaire [22] and biochemistry tests. The covariates for our analyses included sociodemographic characteristics (age, sex, education), routine physical checkup (body mass index [BMI], and blood pressure), lifestyle behaviors (smoking, alcohol drinking, physical activity, fresh fruit intake, vegetable consumption, meat intake), self-reported medical history (diabetes mellitus, heart disease, cerebrovascular disease, respiratory disease including bronchitis, emphysema, asthma, and pneumonia), activities of daily living (ADL), lipid profile, fasting blood glucose, high sensitivity c-reactive protein, and uric acid.

### Assessment of deaths

We ascertained the survival status of included participants in the seventh (2014) and the eighth (2017) wave of the CLHLS survey. The date of death was recorded and confirmed by participants’ family members or local doctors. The survival time for participants was defined as the period from the date of the 2011 survey to the date of death. A ‘lost to follow-up’ status was assigned to those who could not be found and contacted. The data for the participants who survived until the 2017 survey were censored at the time of the 2017 survey.

### Statistical analysis

We summarized the baseline characteristics of included participants by quintiles of SOD activity and MDA levels. We evaluated all-cause mortality by quintiles of SOD activity and MDA levels with Kaplan-Meier survival plots. Equality of death distributions was tested with log-rank tests. We used Cox proportional-hazards models to evaluate the hazard ratios (HRs) and 95% confidence intervals (CIs) by quintiles of SOD and MDA taking the lowest quintile as the reference group. We also evaluated the HRs for mortality by each 10 U/mL increase in SOD activity and each 5 μmol /L increase in MDA. The proportional hazards assumption of the Cox regression model was tested by plotting the log of the cumulative hazard functions against time and checking for parallelism [23]. Because our preliminary analysis showed a significant interaction effect of sex with the association between SOD activity and mortality (*P*-interaction = 0.008), the analyses of SOD activity were carried out in women and men separately.

We applied multivariable Cox regression models to adjust for established and potential risk factors for mortality. We adjusted for age at baseline (continuous), sex (women or men, for MDA) and residence (urban or rural) in the basic analysis model. In the fully-adjusted models we controlled for years of education (0 or ≥ 1 year), frequent vegetable consumption (yes or no), frequent fruit consumption (yes or no), frequent meat consumption (yes or no), frequent physical exercise (yes or no), smoking (current smoker or non-current smoker), alcohol drinking (current drinker or non-current drinker), hypertension (yes or no, confirmed by baseline blood pressure), BMI (continuous), fasting blood glucose (< 7 mmol/L or > =7 mmol/L, based on the criteria for the diagnosis of diabetes mellitus for the Chinese population [24]), total cholesterol (< 6.2 mmol/L or > =6.2 mmol/L, based on the criteria for the diagnosis of hyperlipidemia for the Chinese population [25]), and triglycerides (< 2.3 mmol/L or > =2.3 mmol/L, based on the criteria for the diagnosis of hyperlipidemia for the Chinese population [25]).

We conducted subgroup analyses of the associations of each 10 U/mL increase in SOD and each 5 μmol /L increase in MDA with mortality by age (65–89 years or > =90 years), residence (urban or rural), years of education (0 or >=1 year), smoking status (current or not current), drinking status (current or not current), frequent vegetable consumption (yes or no), frequent fruit consumption (yes or no), and BMI (< 18.5 kg/m^2^, > = 18.5 kg/m^2^, or < 24 kg/m^2^). The interaction effects were tested by including interaction terms in Cox models. As the numbers of participants and deaths in each quintile were too small to provide a precise effect estimate, subgroup analyses for the associations of SOD and MDA with mortality by quintiles were not performed.

We conducted a number of sensitivity analyses to check the robustness of the primary results: 1) additionally adjusting for ethnic group (Han or minority) as the SOD activity may be different by ethnic group [26]; 2) additionally adjusting for frequent milk intake (yes or no) which is a factor that may influence the SOD activity [26]; 3) additionally adjusting for cognitive impairment (yes or no, defined as Mini-Mental State Examination [MMSE] < 24 [27]) for potential confounding effect [28]; 4) additionally adjusting for high sensitive c-reactive protein which is a marker of inflammation [29]; 5) excluding the participants who died in the first year; and 6) excluding participants with a history of diabetes mellitus, heart disease, cerebrovascular disease, or respiratory diseases. A two-tailed *P*-value of less than 0.05 was considered statistically significant. Analyses were completed using Stata version 12.0 (StataCorp LP, College Station, TX, USA).

## Results

### Baseline characteristics

Table 1 presents the baseline characteristics of the participants. The median age of participants was 86 years (interquartile range 76 to 98 years). A total of 997 participants (44.8%) were men and 385 (17.3%) were living in urban areas. The mean SOD activity of included participants was 57.4 U/mL and was lower in men (56.18 U/mL) than in women (58.5 U/mL) (*P* < 0.001). The SOD activity was positively associated with age, with a median age of 83 years for the lowest quintile and 90 years for the highest quintile. The mean MDA level was 5.35 μmol /L (SD: 2.89 μmol /L) with no significant difference between women and men (*P* = 0.24) and was not significantly associated with age (*P* = 0.09).

**Table 1:** Characteristics of participants by quintiles of superoxide dismutase and malondialdehyde

	Superoxide dismutase, U/mL					Malondialdehyde, μmol /L				
	Quintile 1, ~ 50.86	Quintile 2, 50.87~55.53	Quintile 3, 55.54~59.41	Quintile 4, 59.42~63.52	Quintile 5, 63.53~	Quintile 1, ~ 3.56	Quintile 2, 3.57~4.39	Quintile 3, 4.40~5.13	Quintile 4, 5.14~6.20	Quintile 5, 6.21~
No. of participants	445	443	446	445	444	442	446	447	446	442
Age, median (IQR), years	83(73–94)	84(73–96)	87(76–97.3)	88(78–100)	90(80–100)	90(79–100)	87(77–97)	86(76–96)	84-(74–96)	86(74–100)
Female, n(%)	204(45.8)	227(51.2)	227(50.9)	267(60.0)	302(68.0)	253(57.2)	235(52.7)	243(54.4)	253(56.7)	242(54.8)
Residence, n(%)										
Urban	124(27.9)	82(18.5)	50(11.2)	60(13.5)	69(15.5)	82(18.6)	103(23.1)	97(21.7)	66(14.8)	37(8.4)
Rural	321(72.1)	361(81.5)	396(88.8)	385(86.5)	375(84.5)	360(81.4)	343(76.9)	350(78.3)	380(85.2)	405(91.6)
Education time, years										
0	251(57.0)	243(55.2)	281(63.4)	299(68.1)	322(73.2)	302(69.1)	270(61.1)	257(57.8)	255(57.6)	311(71.5)
>=1	189(43.0)	197(44.8)	162(36.6)	140(31.9)	118(26.8)	135(30.9)	172(38.9)	188(42.2)	188(42.4)	124(28.5)
Smoking status, n(%)										
Current	73(17.7)	87(20.0)	87(19.8)	61(13.8)	54(12.4)	69(16.2)	81(19.1)	71(16.4)	79(17.9)	63(14.4)
Not current	340(82.3)	348(80.0)	352(80.2)	382(86.2)	382(87.6)	358(83.8)	344(80.9)	363(83.6)	363(82.1)	375(85.6)
Alcohol drinking status, n(%)										
Current	68(16.3)	83(19.1)	71(16.2)	64(14.5)	40(9.1)	52(12.1)	65(15.3)	74(17.1)	80(18.1)	56(12.7)
Not current	349(83.7)	352(80.9)	367(83.8)	377(85.5)	398(90.9)	379(87.9)	359(84.7)	359(82.9)	361(81.9)	384(87.3)
Frequent vegetable intake, n(%)	191(46.0)	199(45.9)	220(50.5)	196(44.3)	180(41.0)	193(44.8)	211(49.8)	214(49.3)	230(52.8)	139(31.5)
Frequent fruit intake, n(%)	147(35.3)	175 (40.0)	135(30.8)	157 (35.4)	138 (31.2)	128 (29.6)	139 (32.6)	174 (39.9)	181 (41.0)	130 (29.4)
Frequent meat intake, n(%)	305 (73.5)	302(69.1)	301 (69.0)	284 (64.0)	300 (67.9)	264 (61.1)	289 (68.2)	302(69.3)	285 (64.6)	353 (80.0)
Frequent physical activity, n(%)	55 (13.7)	75 (17.7)	68(16.0)	74(17.1)	52(12.1)	60 (14.2)	67 (16.2)	70 (16.5)	74(17.3)	53 (12.4)
Restricted ADL, n(%)	337 (83.2)	328 (77.2)	338 (79.9)	330 (76.7)	342 (80.1)	336 (80.0)	345 (82.5)	329 (76.7)	337 (80.6)	329 (77.4)
BMI, mean (SD), kg/m^2^	21.9 (4.2)	21.7 (4.1)	21.1 (4.1)	21.0 (4.2)	20.6 (4.9)	20.3 (4.3)	21.27 (4.1)	21.3 (4.1)	21.7 (4.9)	21.7 (4.2)
Hypertension, n(%)	166 (40.2)	193 (45.0)	207 (48.7)	201 (46.6)	165 (39.7)	190 (44.6)	178 (42.7)	177 (42.1)	188 (43.9)	198 (46.8)
Glucose, mean (SD), mmol/l	234.8 (39.6)	238.6 (54.8)	236.1 (36.5)	237.9 (35.1)	242.6 (46.6)	235.7 (40.1)	238.7 (43.1)	240.3 (48.3)	240.1 (47.4)	235.3 (35.4)
Total cholesterol, mean (SD), mmol/l	4.2 (1.0)	4.3 (1.0)	4.2 (1.0)	4.3 (1.0)	4.4 (1.0)	4.0 (0.9)	4.2 (1.0)	4.3 (1.0)	4.4 (1.0)	4.5 (1.0)
Triglycerides, mean (SD), mmol/l	1.2(0.9)	1.0(0.6)	0.9(0.5)	1.0(0.6)	0.9(0.5)	0.9(0.5)	0.9(0.6)	1.0(0.7)	1.1(0.7)	1.0(0.7)

IQR: interquartile range; ADL: activity of daily living; NA: not available; BMI: body mass index

### SOD activity and all-cause mortality

During 6 years of follow-up, we documented 858 deaths (women: 538, men: 320), accounting for 46.1% of all participants. Additional file 1: Figure S1 and Additional file 2: Figure S2 present the Kaplan–Meier plots for all-cause mortality by quintiles of SOD activity in women and men, respectively. The log-rank tests suggested that the differences in mortality among different levels of SOD were not statistically significant in women (*P* = 0.51) and in men (*P* = 0.45).

Table 2 presents the association between SOD activity and mortality in women and men. Regarding women, the unadjusted model suggested that the highest quintile was associated with lower but not significant all-cause mortality when compared with the lowest quintile (HR 0.79, 95% CI 0.61 to 1.03). The estimated effects were increased after adjusting for potential confounders. A higher level of SOD activity was associated with a lower risk of mortality (*P*-trend < 0.001). Compared with the lowest quintile in the fully adjusted model, HRs for mortality in the second through fifth quintiles were 0.73 (95% CI 0.53 to 1.02), 0.52(95% CI 0.38 to 0.72), 0.53(95% CI 0.39 to 0.73), and 0.48(95% CI 0.35 to 0.66), respectively. Evaluating the risk of mortality for each 10 U/mL increase in SOD activity yielded an adjusted-HR of 0.82(95% CI 0.74 to 0.92). However, there was no significant association between SOD activity and mortality in men based on analysis by quintiles or evaluation of SOD activity as a continuous variable.

**Table 2:** Association between superoxide dismutase activity and all-cause mortality in women and men

	HR [95% CI] for all-cause mortality		
	Unadjusted model	Basic model ^a^	Fully adjusted model ^b^
Women			
*No. of participants*	1034	1034	822
*No. of events*	537	537	407
No. of person years	3521.9	3521.9	2925.3
Risk at each 10 U/mL increase in SOD activity	0.96 [0.88, 1.05]	0.84[0.77, 0.92] ***	0.82[0.74, 0.92] **
Risk by quintiles			
Quintile 1	1.00(reference)	1.00(reference)	1.00(reference)
Quintile 2	0.93[0.70, 1.22]	0.81[0.61, 1.07]	0.73 [0.53, 1.02]
Quintile 3	0.90 [0.68, 1.19]	0.61 [0.46, 0.81]**	0.52 [0.38, 0.72]***
Quintile 4	0.87 [0.66, 1.13]	0.60 [0.45, 0.79]***	0.53 [0.39, 0.73]***
Quintile 5	0.79 [0.61, 1.03]	0.54 [0.41, 0.71]***	0.48 [0.35, 0.66]***
*P-trend*	*0.07*	*< 0.001*	*< 0.001*
Men			
*No. of participants*	818	818	683
*No. of events*	319	319	249
*No. of person years*	3160.9	3160.9	2715.9
Risk at each 10 U/mL increase in SOD	1.05 [0.94, 1.17]	0.97 [0.86, 1.08]	0.99 [0.86, 1.14]
Risk by quintiles			
Quintile 1	1.00(reference)	1.00(reference)	1.00(reference)
Quintile 2	1.25 [0.90, 1.72]	1.17 [0.84, 1.61]	1.13 [0.77, 1.68]
Quintile 3	1.24 [0.89, 1.71]	1.16 [0.84, 1.61]	1.11 [0.75, 1.66]
Quintile 4	1.02 [0.71, 1.46]	0.74 [0.52, 1.06]	0.74 [0.48, 1.14]
Quintile 5	1.30 [0.90, 1.86]	1.10 [0.77, 1.58]	1.14 [0.74, 1.76]
*P-trend*	*0.40*	*0.90*	*0.64*

HR: hazard ratio; CI: confidence interval; SOD: superoxide dismutase

a. Basic model: adjusted for age (continuous), and residence (urban or rural);

b. Fully adjusted model: additionally adjusted for frequent vegetable consumption (yes or no), frequent fruit consumption (yes or no), frequent meat consumption (yes or not), frequent physical exercise (yes or no), smoking (current smoker, non-current smoker), alcohol drinking (current drinker, non-current drinker), hypertension (yes or no), body mass index (continuous), glucose (< 7 mmol/L or > =7 mmol/L), total cholesterol (< 6.2 mmol/L, > = 6.2 mmol/L), triglycerides(< 2.3 mmol/L or > =2.3 mmol/L), malondialdehyde (continuous)

* *P* < 0.05; **P < 0.01; *** *P* < 0.001

### MDA level and all-cause mortality

Additional file 3: Figure S3 presents the Kaplan–Meier plots for all-cause mortality by quintiles of MDA. The log-rank test suggested no significant differences among quintiles. Cox regression analyses indicated no significant difference in the mortality for each 5 μmol /L increase in MDA (adjusted-HR 0.91, 95% CI 0.79 to 1.04) (Table 3). In the basic model, the quintile 3 had a significantly lower risk of all-cause mortality as compared with the lowest quintile (HR 1.27, 95% CI 1.03 to 1.56), but the estimate was not significant after adjusting for other potential confounders (HR 1.22, 95% CI 0.96 to 1.56).

**Table 3:** Association between malondialdehyde and all-cause mortality

	HR [95% CI] for all-cause mortality		
	Unadjusted model	Basic model ^a^	Fully adjusted model ^b^
*No. of participants*	1853	1853	1505
*No. of events*	857	857	656
*No. of person years*	6686.9	6686.9	5641.2
Risk at each 5 μmol /L increase in MDA	0.96 [0.86, 1.08]	0.96 [0.86, 1.08]	0.91 [0.79, 1.04]
Risk by quintiles			
Quintile 1	1.00(reference)	1.00(reference)	1.00(reference)
Quintile 2	1.02 [0.82, 1.26]	1.12 [0.91, 1.39]	1.11 [0.86, 1.42]
Quintile 3	1.06 [0.86, 1.31]	1.27 [1.03, 1.56]*	1.22 [0.96, 1.56]
Quintile 4	0.85 [0.68, 1.05]	1.11 [0.89, 1.38]	1.08 [0.83, 1.40]
Quintile 5	1.02 [0.82, 1.26]	1.09 [0.88, 1.36]	0.99 [0.77, 1.28]
*P-trend*	*0.46*	*0.51*	*0.79*

HR: hazard ratio; CI: confidence interval; MDA: malondialdehyde

a. Basic model: adjusted for age (continuous), sex (men or women), and residence (urban or rural)

b. Fully adjusted model: additionally adjusted for frequent vegetable consumption (yes or no), frequent fruit consumption (yes or not), frequent meat consumption (yes or no), frequent physical exercise (yes or no), smoking (current smoker, non-current smoker), alcohol drinking (current drinker, non-current drinker), hypertension (yes or no), body mass index (continuous), glucose (< 7 mmol/L or > =7 mmol/L), total cholesterol (< 6.2 mmol/L, > = 6.2 mmol/L), triglycerides(< 2.3 mmol/L or > =2.3 mmol/L), superoxide dismutase (continuous)

*P = 0.05

### Subgroup analyses

**Fig. 2:**
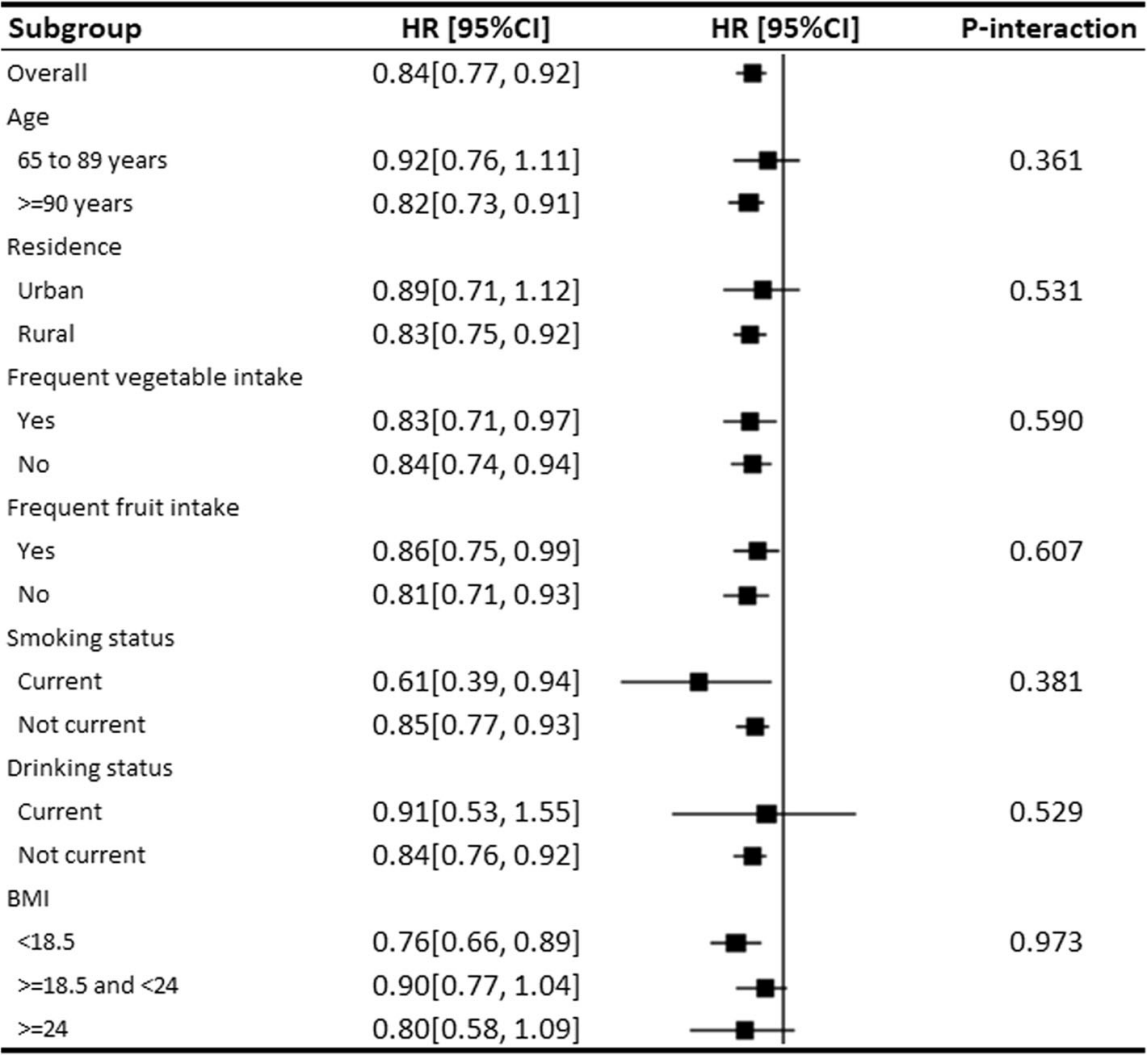
Subgroup analyses for the hazard ratio of all-cause mortality for each 10 U/mL increase in superoxide dismutase activity in women. HR: hazard ratio; CI: confidence interval; BMI: body mass index

The subgroup analysis for the association between SOD activity and mortality by sex showed a significant interaction effect (*P* < 0.001). Subgroup analyses for SOD activity were therefore performed in women and men separately. Figure 2 presents the subgroup analyses for SOD activity in women. We found no significant interaction effects for age, residence, years of education, smoking status, drinking status, frequent vegetable consumption, frequent fruit consumption, and BMI. The subgroup analyses for SOD activity in men and for MDA in all participants are shown in Additional file 4: Table S1 and Additional file 5: Table S2. No significant interaction effects were found regarding the aforementioned factors.

### Sensitivity analyses

Our sensitivity analyses, additionally adjusting for ethnic group, frequent milk intake, cognitive impairment, and high sensitivity c-reactive protein, yielded results largely similar to the primary results (see Additional file 6: Table S3 and Additional file 7: Table S4). There were no material changes in results after excluding the participants who died in the first 6 months and excluding the older adults with a history of diabetes mellitus, heart disease, cerebrovascular disease, or respiratory diseases.

## Discussion

In this prospective cohort study of community-dwelling older adults, we observed a dose-response relationship between blood SOD activity and all-cause mortality in women, but not in men. The association was unlikely to be modified by age, residence, education, smoking, alcohol drinking, vegetable, fruit consumption, and BMI. We did not observe a significant association between MDA level and mortality.

The free radical theory positions an increased production of ROS at the center of age-related cellular injuries [3, 30]. Because SOD enzymes play a key role in regulating cellular ROS levels, decreased SOD activity may increase the vulnerability of cells to oxidative stress and lead to premature death [7]. In *Saccharomyces cerevisiae* [8, 9] and Drosophila models [9], over-expression of SOD protects against oxidative damage and extend life span. Feeding SOD mimetic drugs, such as Euk-8 and MitoQ, increase lifespan in *Drosophila melanogaster* [31]. In humans, the association between SOD activity with life span remains unclear although some observational studies have suggested that high SOD activity is associated with decreased mortality from cancer [14, 15]. In a cohort of 507 healthy Japanese participants aged 40 years or above with an 18-year follow-up, there was no significant association between blood SOD activity and all-cause mortality (HR for the lowest quartile vs. the highest quartile 1.35, 95% CI 0.64 to 2.97) [14]; this failure to detect an association may be a result of the small sample size and low death rate (12.6%) that would have provided inadequate study power to detect a difference. The negative association between SOD activity and mortality agreed with previous studies investigating SOD genes. In a Danish cohort of 1650 older adults (aged 92 to 93 years), individuals with the MnSOD rs4880 C alleles, known to be associated with high SOD2 enzyme activity [32], were associated with decreased mortality (HR for CC vs. CT 0.91, 95% CI 0.86 to 0.97) [33]. A prospective cohort study of 2799 subjects aged > 40 years suggested that the homozygous T allele of rs1041740 was associated with all-cause mortality (HR for TT vs. CC 1.53, 95% CI 1.01 to 2.30) [34].

Perhaps one of the most striking findings of this study was the sex-specific difference in SOD. Consistent with previous studies [35, 36], our results suggested that the average SOD activity was higher in women than in men. A sex-specific difference in the association of SOD activity with mortality was also shown in our analyses. In a Drosophila model, higher SOD activity was far more likely to increase lifespan in females than in males [10], and feeding exogenous antioxidants increased the lifespan of only female flies [31]. The mechanism for the sex-specific differences of SOD on longevity remains unclear. Possible explanations include 1) differences in metabolic rates may lead to different levels of oxidative damage between males and females [37], 2) endocrinological differences by sex may result in different sensitivity to oxidative stress [38], and 3) interactions between the SOD gene with the sex chromosomes [39].

Though in vitro and in vivo studies have suggested that MDA is a stable end product of lipid peroxidation that is associated with aging [12], we did not observe a significant association between MDA level and mortality in older adults. Additional analysis of the relationship between MDA level and SOD activity did not show any significant association. Although the pathway of MDA production by enzymatic processes is well known, its biological functions and its possible dose-dependent dual role have not been fully studied [40].

To the best of our knowledge, this is the first prospective cohort study to evaluate the association of SOD activity, MDA level with mortality in community-dwelling older adults. The strength of this study included the large sample size, careful adjustment for established and potential risk factors, investigation of potential interactions, and robust results of sensitivity analysis.

Our study has several limitations. First, we take single SOD activity test as exposure, which may be unable to present the overall SOD activity level for individual participant because oxidative stress is a dynamic process. However, we focused on the population average SOD level as this study is a population-based observational study. Such method has been commonly used to evaluate the association between SOD activity with other endpoints including cognitive decline, diabetes, hypertension, and cancer mortality. Future studies may consider repeated measure of SOD activity to minimize the potential influence of the dynamic change in SOD though such bias can hardly be fully controlled. Second, despite our best efforts to adjust for established and potential confounders, residual confounding by other unmeasured or unknown factors remains possible. Third, we cannot undertake additional analyses to investigate the type of death because such data were not collected in CLHLS. Fourth, a total of 369 participants (16.6%) were lost to follow-up in this study. However, the potential influence would be minor as the proportions of lost to follow-up were generally balanced among groups and there were no major differences in the main characteristics between those lost to follow-up and others (age: *P* = 0.09, sex: *P* = 0.18, residence: *P* = 0.052; education: *P* = 0.03 (17.8% vs. 22.8%); smoking: *P* = 0.34, alcohol drinking: *P* = 0.41, BMI: *P* = 0.32). Last, this study only explored baseline SOD activity and MDA level, the potential changes in these markers overtime were not adjusted.

## Conclusions

Overall, our analyses indicated that higher baseline plasma SOD activity level was associated with lower overall mortality in older women but not in men. SOD activity may be considered as a prognostic biomarker for mortality in older women. Our findings suggested the importance of integrative approach in the application of different parameters of oxidative stress as potential prognostic biomarkers. Though a causal effect cannot be confirmed due to the observational study design, this study added epidemiological evidence to the free radical theory of aging. Our findings also lend support for interventions to increase the SOD activity, such as exercise and energy-restricted diet [41], to increase longevity.

## Additional files

Additional file 1: **Figure S1.** Kaplan–Meier plot showing the all-cause mortality by quintiles of superoxide dismutase in women (PDF 159 kb)

Additional file 2: **Figure S2.** Kaplan–Meier plot showing the all-cause mortality by quintiles of superoxide dismutase in men (PDF 153 kb)

Additional file 3: **Figure S3.** Kaplan–Meier plot showing the all-cause mortality by quintiles of malondialdehyde (PDF 159 kb)

Additional file 4: **Table S1.** Subgroup analyses for the hazard ratio of all-cause mortality for each 10 U/mL increase in superoxide dismutase activity in men (DOCX 27 kb)

Additional file 5: **Table S2.** Subgroup analyses for the hazard ratio of all-cause mortality for each 5 μmol/L increase in malondialdehyde (DOCX 27 kb)

Additional file 6: **Table S3.** Sensitivity analyses for the association between superoxide dismutase and all-cause mortality (DOCX 30 kb)

Additional file 7: **Table S4.** Sensitivity analyses for the association between malondialdehyde and all-cause mortality (DOCX 29 kb)

## Abbreviations

ADL: Activities of Daily Living
BMI: Body Mass Index
CIs: Confidence Intervals
CLHLS: Chinese Longitudinal Healthy Longevity Survey
HRs: Hazard Ratios
MDA: Malondialdehyde
MMSE: Mini-Mental State Examination
NACDA: National Archive of Computerized Data on Aging
ROS: Reactive Oxygen Species
SOD: Superoxide Dismutase
