# Supplementary material for: Associations between superoxide dismutase, malondialdehyde and all-cause mortality in older adults: a community-based cohort study
Source: BMC Geriatr. 2019 Apr 15;19:104. doi: 10.1186/s12877-019-1109-z (PMC6466801; doi:10.1186/s12877-019-1109-z)
Supplement: Supplementary file 2 — Figure S2. Kaplan–Meier plot showing the all-cause mortality by quintiles of superoxide dismutase in men (PDF 153 kb) [file 12877_2019_1109_MOESM2_ESM.pdf]

Additional file 2

Additional Figure 2. Kaplan–Meier plot showing the all-cause mortality by quintiles of superoxide dismutase in men

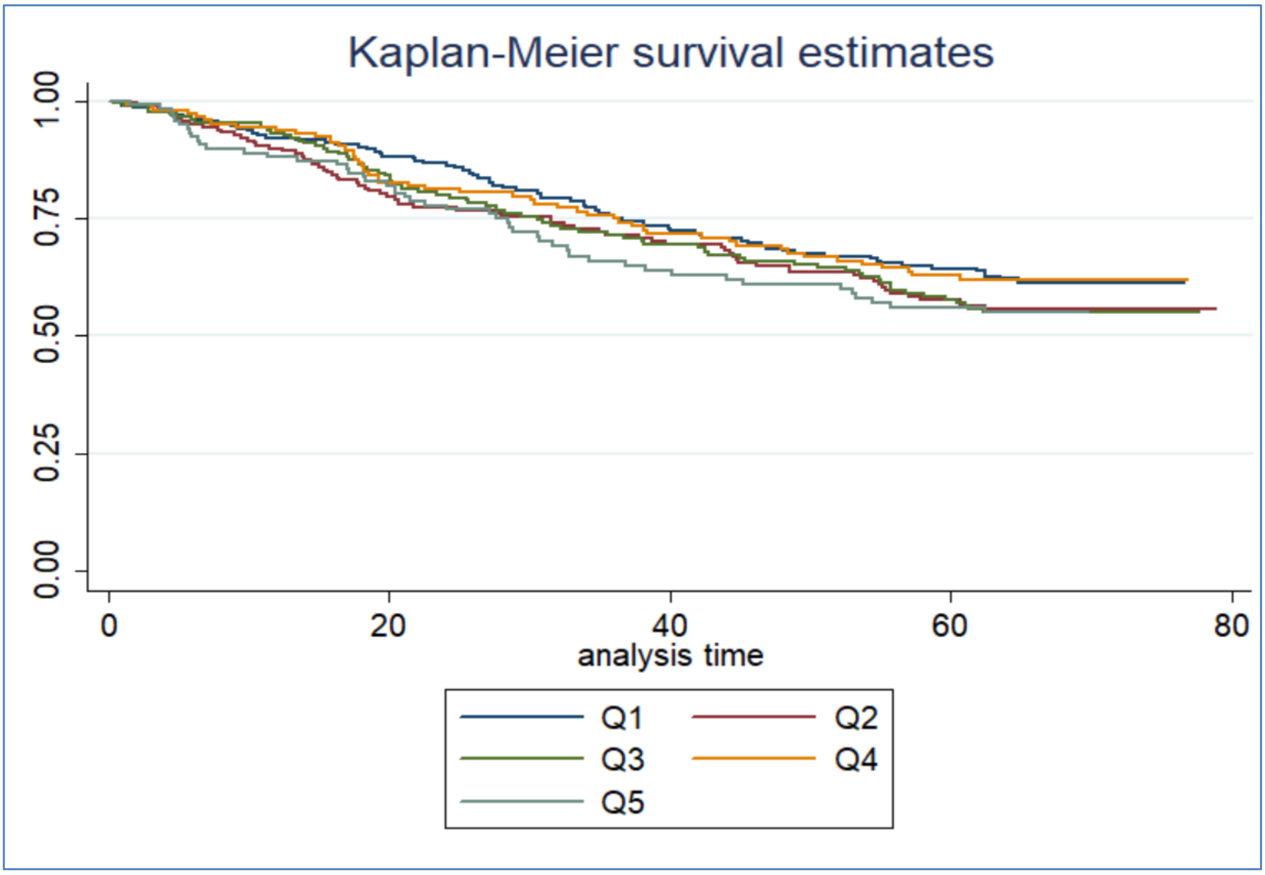

log-rank test p=0.45
