# Supplementary material for: Associations between superoxide dismutase, malondialdehyde and all-cause mortality in older adults: a community-based cohort study
Source: BMC Geriatr. 2019 Apr 15;19:104. doi: 10.1186/s12877-019-1109-z (PMC6466801; doi:10.1186/s12877-019-1109-z)
Supplement: Supplementary file 3 — Figure S3. Kaplan–Meier plot showing the all-cause mortality by quintiles of malondialdehyde (PDF 159 kb) [file 12877_2019_1109_MOESM3_ESM.pdf]

Additional file 3

Additional Figure 3. Kaplan–Meier plot showing the all-cause mortality by quintiles of malondialdehyde

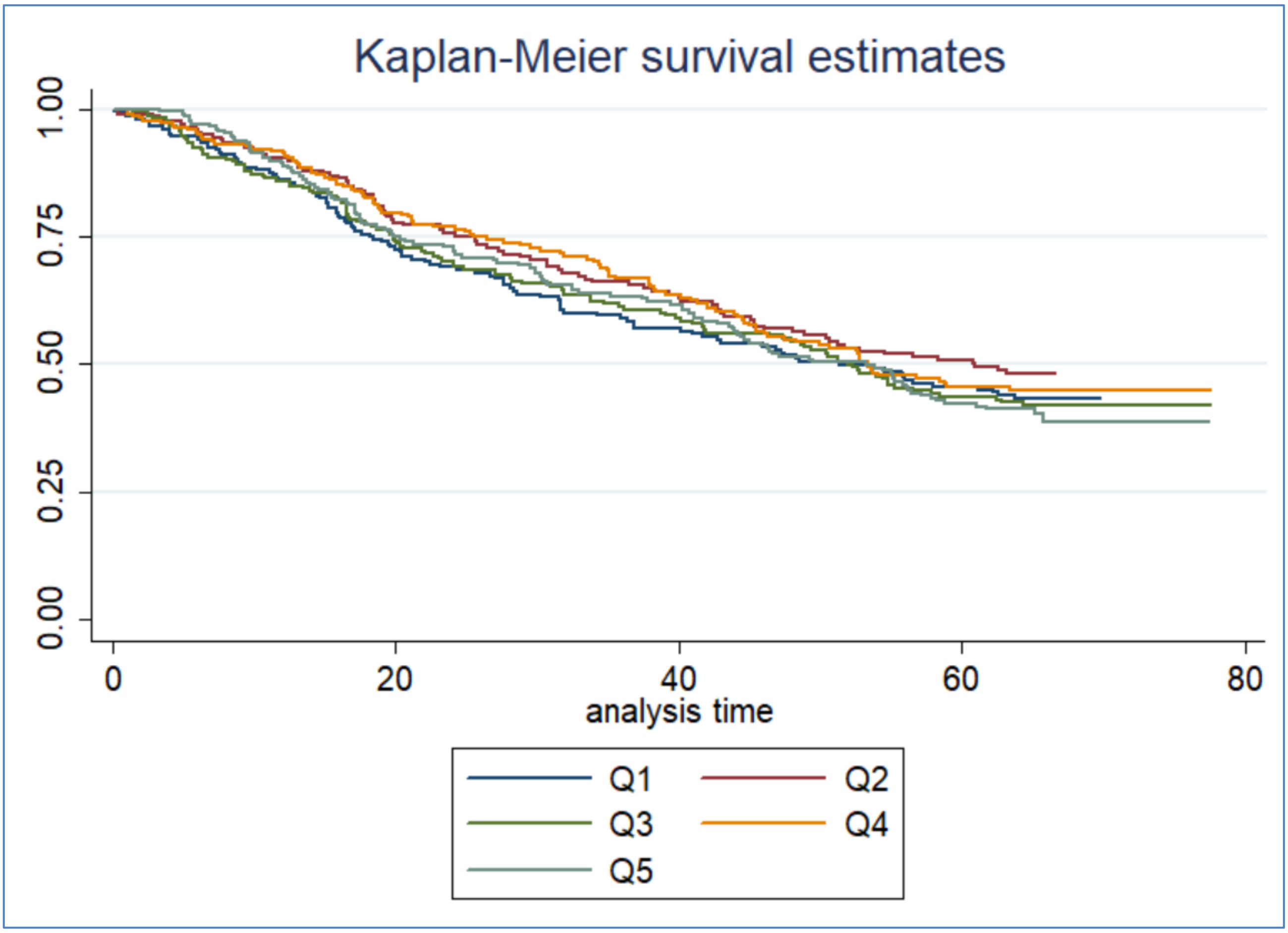

log-rank test p=0.36
