# Supplementary material for: Associations between superoxide dismutase, malondialdehyde and all-cause mortality in older adults: a community-based cohort study
Source: BMC Geriatr. 2019 Apr 15;19:104. doi: 10.1186/s12877-019-1109-z (PMC6466801; doi:10.1186/s12877-019-1109-z)
Supplement: Supplementary file 4 — Table S1. Subgroup analyses for the hazard ratio of all-cause mortality for each 10 U/mL increase in superoxide dismutase activity in men (DOCX 27 kb) [file 12877_2019_1109_MOESM4_ESM.docx]

**Additional file 4**

**Additional Table S1. Subgroup analyses for the hazard ratio of all-cause mortality for each 10 U/mL increase in superoxide dismutase activity in men**

| **Subgroup** | **HR [95%CI]** | **P-interaction** |
| --- | --- | --- |
| Overall | 0.97[0.86, 1.08] |  |
| Age |  |  |
| 65 to 89 years | 1.08[0.92, 1.26] | 0.22 |
| >=90 years | 0.93[0.79, 1.10] |  |
| Residence |  |  |
| Urban | 0.87[0.68, 1.10] | 0.25 |
| Rural | 1.01[0.88, 1.15] |  |
| Frequent vegetable intake |  |  |
| Yes | 0.80[0.62, 1.03] | 0.19 |
| No | 1.03[0.90, 1.19] |  |
| Frequent fruit intake |  |  |
| Yes | 0.98[0.84, 1.15] | 0.92 |
| No | 0.96[0.80, 1.15] |  |
| Smoking status |  |  |
| Current | 1.10[0.85, 1.43] | 0.17 |
| Not current | 0.91[0.80, 1.05] |  |
| Drinking status |  |  |
| Current | 1.07[0.84, 1.36] | 0.38 |
| Not current | 0.94[0.82, 1.08] |  |
| BMI |  |  |
| <18.5 | 0.78[0.61, 1.01] | 0.06 |
| >=18.5 and <24 | 1.02[0.86, 1.20] |  |
| >=24 | 1.29[0.97, 1.71] |  |

HR: hazard ratio; CI: confidence interval; BMI: body mass index

Sensitivity analyses were based on the basic model for primary analysis.
