# Supplementary material for: Associations between superoxide dismutase, malondialdehyde and all-cause mortality in older adults: a community-based cohort study
Source: BMC Geriatr. 2019 Apr 15;19:104. doi: 10.1186/s12877-019-1109-z (PMC6466801; doi:10.1186/s12877-019-1109-z)
Supplement: Supplementary file 5 — Table S2. Subgroup analyses for the hazard ratio of all-cause mortality for each 5 μmol/L increase in malondialdehyde (DOCX 27 kb) [file 12877_2019_1109_MOESM5_ESM.docx]

**Additional file 5**

**Additional Table S2. Subgroup analyses for the hazard ratio of all-cause mortality for each 5 µmol/L increase in malondialdehyde**

| **Subgroup** | **HR [95%CI]** | **P-interaction** |
| --- | --- | --- |
| Overall | 0.96[0.86, 1.08] |  |
| Age |  |  |
| 65 to 89 years | 0.92[0.73, 1.17] | 0.63 |
| >=90 years | 0.99[0.87, 1.12] |  |
| Sex |  |  |
| Women | 0.74[0.58, 0.94] | 0.10 |
| Man | 1.04[0.92, 1.19] |  |
| Residence |  |  |
| Urban | 1.09[0.73, 1.64] | 0.51 |
| Rural | 0.96[0.85, 1.07] |  |
| Frequent vegetable intake |  |  |
| Yes | 0.98[0.80, 1.21] | 0.54 |
| No | 0.97[0.85, 1.10] |  |
| Frequent fruit intake |  |  |
| Yes | 1.02[0.77, 1.34] | 0.61 |
| No | 0.96[0.85, 1.09] |  |
| Smoking status |  |  |
| Current | 0.85[0.49, 1.48] | 0.60 |
| Not current | 0.99[0.88, 1.11] |  |
| Drinking status |  |  |
| Current | 0.63[0.33, 1.24] | 0.15 |
| Not current | 1.00[0.90, 1.13] |  |
| BMI |  |  |
| <18.5 | 1.02[0.80, 1.30] | 0.95 |
| >=18.5 and <24 | 0.98[0.84, 1.14] |  |
| >=24 | 0.97[0.76, 1.24] |  |

HR: hazard ratio; CI: confidence interval; BMI: body mass index

Sensitivity analyses were based on the basic model for primary analysis.
