# Supplementary material for: Associations between superoxide dismutase, malondialdehyde and all-cause mortality in older adults: a community-based cohort study
Source: BMC Geriatr. 2019 Apr 15;19:104. doi: 10.1186/s12877-019-1109-z (PMC6466801; doi:10.1186/s12877-019-1109-z)
Supplement: Supplementary file 6 — Table S3. Sensitivity analyses for the association between superoxide dismutase and all-cause mortality (DOCX 30 kb) [file 12877_2019_1109_MOESM6_ESM.docx]

**Additional file 6**

**Additional Table S3. Sensitivity analyses for the association between superoxide dismutase and all-cause mortality**

|  |  |  | **HR[95% CI]** | |  |  |
| --- | --- | --- | --- | --- | --- | --- |
|  | **Additionally adjusting for ethnic group** | **Additionally adjusting for frequent milk intake** | **Additionally adjusting for cognitive impairment** | **Additionally adjusting high sensitive c-reactive protein** | **Excluding the participants who died in the first six months** | **Excluding the participants with a history of diabetes mellitus, heart disease, cerebrovascular disease, or respiratory diseases** |
| **Women** |  |  |  |  |  |  |
| Risk at each 10 U/mL increase in SOD activity | 0.83[0.75, 0.93] | 0.83[0.74, 0.92] | 0.84[0.75, 0.93] | 0.84[0.75, 0.94] | 0.87[0.77, 0.98] | 0.83[0.74, 0.92] |
| Risk by quintiles |  |  |  |  |  |  |
| Quintile 1 | 1.00(reference) | 1.00(reference) | 1.00(reference) | 1.00(reference) | 1.00(reference) | 1.00(reference) |
| Quintile 2 | 0.75[0.54, 1.03] | 0.73[0.53, 1.01] | 0.77[0.55, 1.07] | 0.72[0.52, 1.00] | 0.78[0.55, 1.11] | 0.73[0.53, 1.02] |
| Quintile 3 | 0.54[0.39, 0.75] | 0.52[0.37, 0.72] | 0.52[0.38, 0.73] | 0.52[0.38, 0.73] | 0.55[0.39, 0.78] | 0.52[0.38, 0.72] |
| Quintile 4 | 0.55[0.40, 0.76] | 0.53[0.38, 0.73] | 0.57[0.41, 0.78] | 0.54[0.39, 0.75] | 0.56[0.40, 0.78] | 0.53[0.39, 0.73] |
| Quintile 5 | 0.49[0.36, 0.67] | 0.48[0.35, 0.65] | 0.50[0.36, 0.68] | 0.49[0.36, 0.67] | 0.55[0.40, 0.77] | 0.48[0.35, 0.66] |
| **Men** |  |  |  |  |  |  |
| Risk at each 10 U/mL increase in SOD | 0.99[0.86, 1.14] | 1.00[0.87, 1.15] | 0.99[0.86, 1.13] | 0.99[0.86, 1.14] | 0.97[0.84, 1.12] | 0.99[0.86, 1.14] |
| Risk by quintiles |  |  |  |  |  |  |
| Quintile 1 | 1.00(reference) | 1.00(reference) | 1.00(reference) | 1.00(reference) | 1.00(reference) | 1.00(reference) |
| Quintile 2 | 1.13[0.76, 1.67] | 1.14[0.77, 1.69] | 1.14[0.77, 1.69] | 1.14[0.77, 1.70] | 1.09[0.73, 1.63] | 1.13[0.77, 1.68] |
| Quintile 3 | 1.11[0.75, 1.65] | 1.13[0.76, 1.68] | 1.11[0.74, 1.65] | 1.12[0.75, 1.68] | 1.07[0.71, 1.62] | 1.11[0.75, 1.66] |
| Quintile 4 | 0.74[0.48, 1.14] | 0.75[0.49, 1.16] | 0.74[0.48, 1.14] | 0.75[0.48, 1.16] | 0.73[0.47, 1.13] | 0.74[0.48, 1.14] |
| Quintile 5 | 1.14[0.74, 1.75] | 1.17[0.76, 1.81] | 1.14[0.74, 1.75] | 1.15[0.74, 1.78] | 1.04[0.66, 1.65] | 1.14[0.74, 1.76] |

HR: hazard ratio; CI: confidence interval; SOD: superoxide dismutase

Sensitivity analyses were based on the fully adjusted model for primary analysis.
