# Supplementary material for: Associations between superoxide dismutase, malondialdehyde and all-cause mortality in older adults: a community-based cohort study
Source: BMC Geriatr. 2019 Apr 15;19:104. doi: 10.1186/s12877-019-1109-z (PMC6466801; doi:10.1186/s12877-019-1109-z)
Supplement: Supplementary file 7 — Table S4. Sensitivity analyses for the association between malondialdehyde and all-cause mortality (DOCX 29 kb) [file 12877_2019_1109_MOESM7_ESM.docx]

**Additional file 7**

**Additional Table S4. Sensitivity analyses for the association between malondialdehyde and all-cause mortality**

|  |  |  | **HR[95% CI]** | |  |  |
| --- | --- | --- | --- | --- | --- | --- |
|  | **Additionally adjusting for ethnic group** | **Additionally adjusting for frequent milk intake** | **Additionally adjusting for cognitive impairment** | **Additionally adjusting high sensitive c-reactive protein** | **Excluding the participants who died in the first six months** | **Excluding patients with a history of diabetes mellitus, heart disease, cerebrovascular disease, or respiratory diseases** |
| Risk at each 5 µmol /L increase in MDA | 0.90[0.78, 1.03] | 0.91[0.79, 1.04] | 0.89[0.77, 1.02] | 0.89[0.77, 1.02] | 0.94[0.82, 1.08] | 0.91[0.79, 1.04] |
| Risk by quintiles |  |  |  |  |  |  |
| Quintile 1 | 1.00(reference) | 1.00(reference) | 1.00(reference) | 1.00(reference) | 1.00(reference) | 1.00(reference) |
| Quintile 2 | 1.10[0.86, 1.41] | 1.11[0.86, 1.42] | 1.09[0.85, 1.39] | 1.11[0.86, 1.42] | 1.09[0.85, 1.42] | 1.11[0.86, 1.42] |
| Quintile 3 | 1.19[0.93, 1.52] | 1.22[0.96, 1.56] | 1.18[0.93, 1.51] | 1.21[0.95, 1.55] | 1.09[0.84, 1.41] | 1.22[0.96, 1.56] |
| Quintile 4 | 1.06[0.82, 1.37] | 1.08[0.83, 1.40] | 1.05[0.81, 1.35] | 1.07[0.83, 1.39] | 1.07[0.82, 1.40] | 1.08[0.83, 1.40] |
| Quintile 5 | 0.96[0.75, 1.24] | 0.99[0.77, 1.27] | 0.95[0.74, 1.22] | 0.97[0.76, 1.25] | 1.03[0.80, 1.33] | 0.99[0.77, 1.28] |

HR: hazard ratio; CI: confidence interval; MDA: malondialdehyde

Sensitivity analyses were based on the fully adjusted model for primary analysis.
